# Resilience, perceived danger and work attendance of Sudanese healthcare workers in war zones: the April 2023 armed conflict

**DOI:** 10.1017/gmh.2026.10242

**Published:** 2026-06-18

**Authors:** Maryam Mohamed, Sami Hassan, Abubakr Mahmoud, Laila Osman, Huda Ahmed, Alameen Abass, Elmoez Mhadey, Elharith Abdelwahid, Hiba Hamad, Mohamed Osman

**Affiliations:** 1 University of Khartoum Faculty of Medicine, Khartoum, Sudan; 2 Student association for medical education and research (SAMER), Khartoum medical students association (kMSA), Khartoum, Sudan; 3 Karary University, Omdurman, Sudan; 4 UMST, Khartoum, Sudan

**Keywords:** resilience, sense of danger, work attendance, armed conflict, Sudan

## Abstract

Traumatic war events can impair healthcare workers’ psychological well-being. We assessed resilience, attendance and perceived sense of danger among Sudanese healthcare workers in active conflict zones. A cross-sectional study (January–March 2025) was conducted in four Omdurman public hospitals involving doctors, nurses, lab technicians and pharmacists. We used a self-administered questionnaire assessing sociodemographics, trauma, resilience (Connor-Davidson Resilience Scale-10), sense of danger (Solomon and Prager) and attendance. Data were analyzed using SPSS v27 (*p* < 0.05). Among 325 participants, 60% reported property damage, 49% family injury and 41% security escalations. Attendance was higher among males, single workers, those with crisis training and without personal or family injuries. Median resilience score was 24.00 (IQR = 12.00); 41.23% had low resilience, associated with male gender, religiosity, crisis training, full attendance and hospital assignment. Median sense of danger was high (14.0), linked to security escalations and lack of group activities. Resilience and sense of danger showed a weak positive correlation (rho = 0.178, *p* = 0.001). Healthcare workers in conflict zones exhibited low resilience and high perceived danger, influenced by gender, crisis training and security. Urgent mental health and safety interventions are needed to sustain this critical workforce.

## Impact statement

The armed conflict that erupted in Sudan in April 2023 has caused a severe humanitarian crisis, devastating the healthcare system and leaving only a fraction of hospitals operational in conflict zones. While the physical destruction of infrastructure is visible, the psychological toll on the healthcare workers (HCWs) who remain to treat the wounded is often overlooked. This study provides a rare and critical assessment of the mental resilience and perceived sense of danger among doctors, nurses and pharmacists working directly on the frontlines in Omdurman. Our findings reveal that while work attendance remained relatively high due to a sense of duty, the workforce is operating under extreme duress, with over 70% reporting a high sense of danger and nearly half exhibiting low psychological resilience. Crucially, we identified that previous crisis management training significantly improved attendance and resilience, yet few workers had received it. The wider impact of this research lies in its ability to guide immediate humanitarian interventions. By identifying specific vulnerabilities, such as the lack of crisis training and the correlation between security escalations and mental distress, this study offers a roadmap for policymakers, NGOs and hospital administrators. It highlights that sustaining a functioning health system during war requires more than just medical supplies; it requires urgent, targeted psychosocial support and safety training for the workforce. Protecting these essential workers is vital not only for their well-being but for ensuring the continuity of life-saving care for the entire population during instability.

## Introduction

Mental health is defined by the World Health Organization (WHO) as a state of well-being that allows individuals to handle stress, use their abilities, work and learn effectively and contribute to society (World Health Organization [WHO], 2022). Stressful and traumatic situations pose risks to this state. The WHO estimated that 10% of people exposed to trauma will be severely affected (Murthy and Lakshminarayana, 2006). Armed conflicts in particular disrupt all citizen’s lives, their mental health and health care systems. Moreover, the emerging threat of weapons of mass destruction (nuclear, biological and chemical) creates serious psychological burdens due to their catastrophic impacts on humanity, ecosystems and global stability. Such events can lead to long-term mental health problems, including trauma, anxiety and depression, complicated by limited access to mental health services. Studies have also stated a correlation between exposure to the atomic bombings of Hiroshima and Nagasaki and severe intellectual or developmental impairments (Ahmed and Al Diab Al Azzawi, 2024).

Healthcare Workers (HCWs), being an essential workforce in critical times, are prone to mental health disturbances even during their standard working conditions. Studies conducted between 2020 and 2022 reported that approximately one quarter of HCWs worldwide experience symptoms of anxiety, depression and burnout (WHO, 2024). In conflict zones, these stressors are exacerbated. A study in Syria showed that HCWs had worked for 6.5 h longer than average while treating double the number of patients(Abdelrahman and Haar, 2021). In Gaza, they had to operate under constant threat of violence while navigating the limited supplies, including anesthesia (Abed Alah, 2024). Another study demonstrated high levels of PTSD among Palestinian HCWs, reaching up to 89% (Abu-El-Noor et al., 2018). Other mental health effects of conflicts include anxiety, depression and burnout. Close results were obtained in other conflict settings too, such as Yemen, Afghanistan, Congo and Pakistan (Merlin, 2010; Baker and Heisler, 2015; Rubenstein, 2015; Monaghan, 2017; Afzal and Jafar, 2019).

In light of these daunting challenges, individual resilience – defined as the ability to recover from adversity and gain strength through the recovery process (Yates et al., 2015) remains a key factor influencing functionality and coping with traumatic events. Within healthcare systems, resilience is crucial for maintaining services during crises. A study in Israel found that those who reported fully to work had higher resilience levels (Sberro-Cohen et al., 2023). Factors shown to foster resilience include self-protection measures, internal coping mechanisms such as faith and patriotism, and external support such as international assistance and training (Witter et al., 2017).

Another factor affecting resilience among healthcare workers is the perceived sense of danger, a feeling of being attacked. This has been found to reduce resilience, increase stress levels and contribute to higher rates of absenteeism (Kimhi et al., 2018; Sberro-Cohen et al., 2023).

Since 2018, Sudan has witnessed a period of instability following the overthrow of Omar al-Bashir, which was followed by increasing tensions between the Rapid Support Forces and the national military that eventually escalated into war on April 15, 2023. The conflict has caused around 40,000 injuries and displaced about 11.5 million people, with 6.6 million requiring health support (Elkahlout and Milton, 2022; WHO, 2026). The impact has been even greater on Sudan’s already fragile healthcare system, with about 70% of hospitals forced to close due to looting and destruction (Dafallah et al., 2023). The World Health Organization’s Surveillance System for Attacks on Health Care documented 201 attacks, resulting in 1,858 deaths and 490 injuries. At the same time, outbreaks of diseases such as cholera, malaria and measles have increased, further burdening the already critical situation (WHO, 2026). These circumstances place a significant psychological burden on healthcare professionals, increasing the risk of stress, burnout and other mental health conditions. This raises serious concerns about their mental well-being and the sustainability of the healthcare system.

Literature examining the mental health of healthcare workers in conflict settings is growing, with many documenting high levels of psychological distress. Yet most studies have focused on regions such as Syria, the Gaza Strip and Yemen. Leaving a significant gap in the literature regarding the mental health of healthcare workers in Sudan. In particular, limited attention has been given to how resilience interacts with contextual stressors such as the perceived sense of danger among healthcare workers operating in active conflict environments. Grasping these factors allows better understanding of vulnerabilities and aids in supporting healthcare workers’ mental well-being. In addition to improving the functionality of the entire healthcare system.

Hence comes the purpose of our research: to highlight the struggle of Sudanese healthcare workers and study the factors that might potentially improve their resilience or decrease it and what role the perceived sense of danger plays in this issue.

## Method

### Study design and setting

We conducted a hospital-based cross-sectional study among healthcare professionals in Khartoum State, Sudan. This study was designed and is reported in strict adherence to the Strengthening the Reporting of Observational Studies in Epidemiology (STROBE) statement to ensure transparency and methodological integrity (Vandenbroucke et al., 2007).

The STROBE framework was applied across all stages of manuscript preparation:

*Methodological transparency*: We have clearly operationalized the study’s setting, participant eligibility and the temporal context of the April 2023 conflict to ensure the replicability of our findings.

*Variable specification*: All outcomes, specifically resilience, perceived danger and work attendance, are defined alongside the psychometric properties of their respective instruments.

*Bias mitigation and contextualization*: Consistent with STROBE Item 9, we explicitly address potential sources of bias, including non-response bias and the “survivor bias” inherent in surveying frontline staff during an active conflict, and we have interpreted our findings within these constraints.

*Analytical rigor*: We provide a transparent account of the statistical methods used, including the justification for non-parametric testing and the handling of multivariable models, ensuring that the results follow a logical and verifiable progression from the initial research questions. This cross-sectional study was conducted from January to March 2025. The research was carried out in four major public hospitals in Omdurman city, Khartoum state: Al Nau Teaching Hospital, Alsaudi Teaching Hospital, Albuluk Teaching Hospital and Bir Alwaldeen Teaching Hospital.

The four hospitals were selected using purposive sampling. These facilities were the only major public hospitals that remained operational in Khartoum State during the ongoing armed conflict. Their continued functionality and proximity to active military clashes made them uniquely appropriate for investigating resilience and perceived danger among healthcare workers in frontline war settings.

This study aimed to answer the following research questions:

- What are the levels of resilience, perceived sense of danger and work attendance among healthcare workers in conflict zones?
- What factors predict work attendance during wartime?
- What demographic and occupational factors are associated with resilience?
- What factors are associated with a perceived sense of danger?
- Is there an association between resilience and perceived sense of danger?

### Study population

Eligible participants were identified through official departmental rosters obtained from hospital administration. Department heads facilitated contact with healthcare professionals during working hours. The research team approached eligible staff members individually, explained the study objectives and invited voluntary participation. No incentives were provided.

Healthcare professionals were eligible if they had been working in the selected hospitals for at least 1 month during the ongoing war period (April 2023 onward). The inclusion criterion of at least 1 month of continuous service during the conflict was established to ensure that participants had sufficient and meaningful exposure to the operational challenges and security risks of the wartime healthcare environment. While the conflict has been ongoing for approximately 2 years, a higher threshold (e.g., 6 months or 1 year) would have risked excluding a significant portion of the current workforce, including newly graduated medical staff and reassigned personnel who have been vital to maintaining hospital operations during recent escalations. This one-month window thus captures the experiences of the contemporary frontline workforce while ensuring they have navigated at least one full cycle of the conflict’s current intensity.

#### Eligibility criteria

Inclusion criteria:

- Licensed doctors, nurses, pharmacists and laboratory technicians
- Employed in selected hospitals for ≥1 month during the conflict
- Aged ≥18 years
- Provided informed consent

Exclusion criteria:

- Non-medical volunteers
- Unregistered house officers
- Individuals unable to provide consent

### Sample size and sampling technique

A stratified random allocation was employed to ensure proportional representation of healthcare professionals across the four hospitals included in the study. This approach was chosen due to the heterogeneous distribution of staff among the hospitals, thereby enhancing the generalizability and representativeness of the findings.

#### Stratification and population distribution

The total population of eligible healthcare professionals (*N* = 2,030) was stratified by hospital as follows:

- Alnau Hospital: 700 professionals
- Alsaudi Hospital: 440 professionals
- Albuluk Hospital: 540 professionals
- Bir Alwaldeen Hospital: 350 professionals

The required sample size was calculated using the standard formula for proportions with a 95% confidence level and 5% margin of error:

$n=\left(Z^{2}*p*\left(1-p\right)\right)/e^{2}=\left(1.96^{2}*0.5*\left(1-0.5\right)\right)/0.05^{2}=384$ .

Given the finite target population (*N* = 2,030), a finite population correction (FPC) was applied:

$$n\_\text{adjusted}=n/\left(1+\left(n-1\right)/N\right)=384/\left(1+383/2030\right)\approx 322.$$

Therefore, the minimum required sample size was 322 participants.

To ensure proportional representation across hospitals and to compensate for potential non-response or incomplete questionnaires, the adjusted sample size was proportionally allocated across the four hospitals based on staff distribution. Fractional allocations were rounded to the nearest whole number, resulting in a planned target of 326 participants.

The final number of completed and eligible responses was 325, slightly exceeding the minimum required adjusted sample size (322). This small difference reflects rounding during proportional allocation and minor oversampling to preserve representativeness.

#### Proportional allocation by stratum

The adjusted sample size was distributed proportionally across the four hospitals using the formula:

$$n\_i=\left(N\_i*n\_\text{adjusted}\right)/N.$$

Resulting in the following allocations:

- Alnau (700/2030 × 322) ≈ 111
- Alsaudi (440/2030 × 322) ≈ 70
- Albuluk (540/2030 × 322) ≈ 86
- Bir Alwaldeen (350/2030 × 322) ≈ 55

Total ≈ 322.

On the hospital level, participants were selected conveniently due to the escalating events and the sensitivity of hospital staff personnel information. Potential selection bias introduced by this adjustment is acknowledged as a limitation.

### Data collection and management

Data were collected using a self-administered online questionnaire adopted from previous similar studies and validated scales (Solomon and Prager, 1992; Connor and Davidson, 2003; Sberro-Cohen et al., 2023). While the CD-RISC-10 and the Sense of Danger Scale are validated international instruments, the authors acknowledge the importance of contextual validity in conflict-affected populations. The English versions were utilized as English is the primary medium of instruction in Sudanese medical and healthcare education; therefore, the study population (doctors, pharmacists and senior nurses) possessed a high level of professional English proficiency. To ensure linguistic and conceptual clarity, the survey was pre-tested with a pilot group of 20 healthcare workers from the target hospitals. Participants reported that the items were easily understood and culturally relevant to their experiences of the current crisis. Furthermore, the internal consistency in this study (Cronbach’s alpha of 0.92 for CD-RISC-10 and 0.85 for the Sense of Danger Scale) suggests that the instruments maintained high reliability within this specific Sudanese professional cohort.

Participants were approached in their workplaces during regular duty hours. The research team visited hospitals and invited eligible health care workers to participate after providing a brief explanation of the study’s purpose and ensuring voluntary participation. Participants were provided with the Google Form version of the questionnaire and given the option to fill it in person or later through their WhatsApp or Telegram. All questionnaires were anonymous. The questionnaire consisted of five sections:

*Sociodemographic*: Included questions about general background information and professional status.

*Exposure to terrorist events*: Nine questions about past experiences with terrorism, including damage to property, physical or psychological injuries, exposure at previous workplaces and utilization of support services. (Scholte et al., 2004; Sberro-Cohen et al., 2023).

*Individual resilience*: Was measured using the 10-item Connor–Davidson Resilience Scale (CD-RISC-10), a widely validated instrument assessing psychological adaptability to stress. Each item is scored on a 5-point Likert scale (0–4), with total scores ranging from 0 to 40. The English version used in this study. In the current sample, internal consistency was excellent (Cronbach’s *α* = 0.92).

*Sense of danger*: Perceived sense of danger was assessed using the six-item Solomon and Prager Sense of Danger Scale. Items are rated on a 5-point Likert scale (0–4), producing higher scores for greater perceived danger. The English version of the scale was used. In the present study, internal consistency was good (Cronbach’s *α* = 0.85).

*Work attendance*: Was assessed using three structured self-report items classifying attendance as full, partial or absent during wartime conditions and additional questions about factors that made it difficult or easier to report to work (Yates et al., 2015; Sberro-Cohen et al., 2023).

The reliability coefficients (Cronbach’s *α*) for each scale were derived from the current study sample.

### Ethical consideration

Ethical approval was obtained from the Medical Research Ethics Committee of the University of Al Gadarif (Approval No: GU/FM/REC/Q3.7.25.6). The study adhered to the Declaration of Helsinki principles.

Participation was voluntary. Electronic informed consent was obtained before accessing the questionnaire. Participants were informed of their right to withdraw at any time without consequence. No identifying information was collected, and all data were stored securely with restricted access.

### Statistical analysis

Data were analyzed using SPSS software, version 27. Continuous outcomes (resilience and sense-of-danger scores) were found to be non-normally distributed and are therefore summarized as medians with interquartile ranges (IQR), while categorical variables, including sociodemographics and work attendance levels, are presented as frequencies and percentages.

The specific analyses used to address the research questions were as follows:

*Levels of primary variables*: Descriptive statistics and tertile cutoffs were used to classify resilience and sense of danger levels.

*Predictors of work attendance*: Associations between categorical factors (e.g., gender, injury status or professional role) and work attendance were evaluated using the chi-square test or Fisher’s exact test when expected cell counts were less than five.

*Factors associated with resilience and sense of danger*: The Mann–Whitney *U* test was employed for predictors with two levels. The Kruskal–Wallis H test was used for predictors with three or more levels, followed by Bonferroni-adjusted post hoc comparisons to identify specific group differences.

*Relationship between resilience and perceived danger*: This association was evaluated using Spearman’s rank-order correlation. The coefficient of determination was also calculated to determine the shared variance between the two variables.

Two multivariable linear regression models were constructed (one for resilience and one for sense of danger) to identify significant predictors based on theoretical considerations. All necessary model assumptions, including linearity, homoscedasticity, absence of multicollinearity and independence of residuals, were tested and met. Statistical significance for all analyses was set at *α* = 0.05.

## Results

### Sociodemographic characteristics

A total of 325 healthcare workers were included in the study, slightly exceeding the minimum required sample size. Participants were predominantly young (mean age 28.3 ± 4.9 years), female (57.2%) and single (76.3%). Most lived with family (83.7%) and reported low monthly income (<500,000 SDG; 85.2%). Detailed demographic characteristics are presented in Table 1.

**Table 1.** Demographics Table 1. Long description.

Variable	*n*	%
Age		
18–40	314	96.6%
41 and above	11	3.4%
Gender		
Male	139	42.8%
Female	186	57.2%
Marital status		
Single	248	76.3%
Married	71	21.8%
Divorced	3	0.9%
Widowed	3	0.9%
Do you have dependents in your household?		
Yes	138	42.5%
No	187	57.5%
Children’s age		
Less than 2 years	35	10.8%
2–10	69	21.2%
11–17	28	8.6%
18+	15	4.6%
No children	206	63.4%
Perceived religiosity		
Low	21	6.5%
Moderate	252	77.5%
High	52	16.0%
Nationality		
Sudanese	323	99.4%
Non-Sudanese	2	0.6%
Living arrangements		
Alone	40	12.3%
In dorms	13	4.0%
With family	272	83.7%
Monthly income in SDG		
Less than 500,000	277	85.2%
500,000–1,000,000	40	12.3%
More than 1,000,000	8	2.5%

House officers (27.7%) and medical officers (25.5%) constituted the largest professional groups. Notably, only 39.1% of participants had received crisis management training, indicating limited preparedness for conflict-related challenges. Additional details on professional profiles are provided in Table 2.

**Table 2.** Work related demographics Table 2. Long description.

Variable	*n*	%
Occupation		
Consultant	4	1.2%
House officer	90	27.7%
Lab technician	21	6.5%
Medical officer	83	25.5%
Nurse	62	19.1%
Pharmacist	16	4.9%
Registrar	44	13.5%
Specialist	5	1.5%
The field of specialty for consultants, specialists and registrars*		
Internal medicine	8	2.5%
General surgery	8	2.5%
Orthopedic surgery	4	1.3%
Pediatrics	11	3.5%
Obstetrics and gynecology	12	3.8%
Radiology or neurology	2	0.6%
Hospital		
Alnau	111	34.2%
Albuluk	86	26.5%
Alsuadi	70	21.5%
Bir Alwaldeen	58	17.8%
Transportation to work		
Public transportation	186	57.2%
Hospital shuttle services	82	25.2%
Private vehicle	45	13.8%
Walking/Bicycle	12	3.7%
Crisis management training		
Yes	127	39.1%
No	198	60.9%

* There are eight missing responses in the field of specialty.

### Exposure to terrorist events and use of support services

Exposure to conflict-related events was substantial. A majority of participants reported property damage (60%), while nearly half experienced family injury (49%) and 41% reported security escalations at their workplace.

Despite this high level of exposure, utilization of support services was low. Only small proportions of participants reported engaging in personal support (12%), group activities (18%) or community support services (12%), suggesting limited uptake of available psychosocial support mechanisms. Detailed data are summarized in Supplementary Table S1.

### Levels of resilience, perceived danger and work attendance

The analysis of primary variables revealed the following levels among healthcare workers:

Healthcare workers demonstrated low resilience and high perceived danger overall. The median resilience score was 24.0 (IQR = 12.0), with 41.2% classified as having low resilience.

Perceived sense of danger was high, with a median score of 14.0 (IQR = 7.0) and over 70% of participants falling into the high category.

Despite these conditions, work attendance remained relatively high, with 66.8% of participants maintaining full attendance during wartime.

### Factors associated with work attendance during wartime

Work attendance was significantly associated with several demographic and situational factors. Attendance was higher among males compared to females (74.8% vs. 60.8%, *p* = 0.011) and among single participants (*p* = 0.003).

Participants who experienced physical injury (49.3% vs. 71.3%, *p* = 0.001) or had an injured family member (57.9% vs. 75.3%, *p* = 0.001) were less likely to maintain attendance.

Professional role was also significant (*p* = 0.027), with the highest attendance observed among medical officers (75.9%) and house officers (73.3%), and the lowest among pharmacists (37.5%).

Importantly, participants with prior crisis management training demonstrated higher attendance compared to those without training (74.8% vs. 61.6%, *p* = 0.014). Attendance also varied significantly across hospitals (*p* = 0.001), reflecting potential institutional or contextual differences. Detailed data about attendance are summarized in Table 3.

**Table 3.** Attendance at work during wartime Table 3. Long description.

Variable	Absenteeism (full or partial)% (N)	No absenteeism %(N)
Overall attendance	33.23% (108)	66.77% (217)
Gender		
Male	25.2% (35)	74.8% (104)
Female	39.2% (73)	60.8% (113)
Marital status		
Single	29.0% (72)	71.0% (176)
Married	45.1% (32)	54.9% (39)
Divorced	33.3% (1)	66.7% (2)
Widowed	100.0% (3)	0.0% (0)
Occupation		
Consultant	50.0% (2)	50.0% (2)
House officer	26.7% (24)	73.3% (66)
Lab technician	47.6% (10)	52.4% (11)
Medical officer	24.1% (20)	75.9% (63)
Nurse	40.3% (25)	59.7% (37)
Pharmacist	62.5% (10)	37.5% (6)
Registrar	34.1% (15)	65.9% (29)
Specialist	40.0% (2)	60.0% (3)
Hospital		
Alnau	45.0% (50)	55.0% (61)
Albuluk	9.3% (8)	90.7% (78)
Alsuadi	51.4% (36)	48.6% (34)
Bir Alwaldeen	24.1% (14)	75.9% (44)
Crisis management training		
Yes	25.2% (32)	74.8% (95)
No	38.4% (76)	61.6% (122)
Physically injured by terrorism		
Yes	50.7% (34)	49.3% (33)
No	28.7% (74)	71.3% (184)
Family member injured by terrorism		
Yes	42.1% (67)	57.9% (92)
No	24.7% (41)	75.3% (125)

### Factors associated with and predicting resilience

Non-parametric analyses identified significant associations between resilience and demographic/work-related factors (Supplementary Table S2).

Resilience varied significantly across demographic and occupational factors. Males demonstrated higher resilience than females (*p* < 0.001), and participants without children were more resilient than those with children (*p* = 0.024). Higher levels of religiosity were also associated with increased resilience (*p* = 0.006).

Professionally, resilience differed by role (*p* < 0.001), with specialists and medical officers showing higher levels compared to nurses and allied health staff. Significant variation was also observed across hospitals (*p* < 0.001).

Participants who had received crisis management training (*p* = 0.002) and those maintaining full attendance (*p* = 0.001) exhibited higher resilience.

In multivariable analysis, resilience was significantly associated with work attendance (*p* < 0.001), professional role (*p* = 0.001), hospital (*p* < 0.001), crisis training (*p* = 0.004), participation in group activities (*p* = 0.004), use of personal support (*p* = 0.012), age (*p* = 0.048) and perceived sense of danger (*p* < 0.001). The model explained 30.6% of the variance in resilience scores (*R* ^2^ = 0.306). Table 4 presents the results of the linear regression analysis predicting resilience scale scores.

**Table 4.** Summary of linear regression analysis predicting resilience scale scores Table 4. Long description.

Variable	B	SE	*β*	*t*	*p*
Age groups	5.227	2.628	0.109	1.989	0.048
Job profile	1.315	0.377	0.191	3.487	0.001
Level of work attendance	3.516	0.972	0.191	3.619	<0.001
Hospital you are working in	−1.859	0.409	−0.237	−4.548	<0.001
Training in crisis management	−2.644	0.923	−0.149	−2.863	0.004
Used personal support talks	3.688	1.463	0.139	2.521	0.012
Participated in group activities	3.986	1.367	0.175	2.917	0.004
Sense of danger	0.379	0.094	0.204	4.029	<0.001

*Note:* B: unstandardized regression coefficient; SE: standard error; *β*: standardized regression coefficient; *t*: *t*-statistic; *p*: *p*-value.

### Factors associated with and predicting perceived sense of danger

The associations between sense of danger and various work-related and personal factors are summarized in Supplementary Table S3.

Perceived sense of danger differed significantly across occupational groups (*p* = 0.001), with pharmacists and laboratory technicians reporting higher levels compared to other professions. Differences were also observed across hospitals (*p* = 0.037).

Exposure to security escalation events was significantly associated with higher perceived danger (*p* = 0.047). In contrast, participants who engaged in group activities reported lower levels of perceived danger (*p* = 0.017), suggesting a potential protective effect of social support.

Multivariable analysis showed that higher resilience was associated with increased perceived danger (*p* < 0.001), while exposure to security escalation events was associated with lower perceived danger (*p* = 0.021). The model explained 14.1% of the variance (*R* ^2^ = 0.141). Table 5 presents the results of the linear regression analysis predicting sense of danger scale scores.

**Table 5.** Summary of linear regression analysis predicting sense of danger scale scores Table 5. Long description.

Variable	B	SE	*β*	*t*	*p*
Exposure to security escalation events	−1.344	0.58	−0.142	−2.317	0.021
Resilience scale	0.136	0.034	0.252	4.029	<0.001

*Note:* B: unstandardized regression coefficient; SE: standard error; *β*: standardized regression coefficient; *t*: *t*-statistic; *p*: *p*-value.

### Relationship between resilience and sense of danger

A Spearman’s rank-order correlation examining the relationship between resilience and the perceived sense of danger revealed a weak, positive and statistically significant correlation between the two variables, rho (325) = 0.178, *p* = 0.001. The coefficient of determination (rho-squared) was 0.0317, suggesting that the two variables shared approximately 3.17% of their variance.

## Discussion

We analyzed resilience, perceived sense of danger and work attendance during wartime, looking to pinpoint the factors among healthcare professionals (HCPs) in the only operational hospitals within Khartoum state during the fighting.

Healthcare workers’ attendance in conflict areas is influenced by both demographic and situational factors. In our study, the overall attendance was good, with 66.77% of participants maintaining full attendance. Better attendance was noted among males and single individuals, likely due to lower caregiving responsibilities and personal safety concerns. Similar trends have been documented in Yemen and Sierra Leone (Khader et al., 2015; Wurie et al., 2016). Although two-thirds of participants maintained full attendance, this should not be interpreted as evidence of robust workforce resilience. In conflict settings, attendance may reflect economic necessity, professional duty or lack of viable alternatives rather than psychological strength. Given potential survivor bias, attendance likely represents the response of those who were able to remain rather than the experience of the entire healthcare workforce.

Those who did not suffer personal or familial injuries within the context of the conflict were more likely to attend, illustrating the ways violence directly affects workforce participation. Such findings have been documented in Syria, where personal and familial trauma diminished healthcare workers’ capacities to report to work (Fouad et al., 2017; Witter et al., 2017).

The higher absenteeism at Alsaudi Hospital may stem from geographic location, administrative issues or a combination of both. Previous research has linked high-risk area facilities with poor staff attendance and high vacancy rates (Pavignani and Colombo, 2009).

In Uganda, studies noted physicians maintained a higher presence during crises, so it may be that medical and house officers perceived stronger professional commitment or indispensability when compared with other cadres. They certainly showed better attendance than most (Tashobya et al., 2006).

The median resilience score for participants is concerningly low since 41% demonstrated poor resilience. This supports previous research from conflict-perturbed health systems within Gaza, Libya, Israel and Northern Ethiopia, which documented the enduring impact of insufficient security and relentless working conditions on the psychological functioning and well-being of healthcare personnel (Abugraga et al., 2023; Alnassar et al., 2023; Sberro-Cohen et al., 2023).

The significant number of participants experiencing direct violence, such as physical injuries and damage to their property, was a key contributing factor. Resilience, as expected, was strongly associated with numerous demographic and occupational factors, including gender, parental status, spirituality, professional rank and prior exposure to crisis management training. Among these, males, non-parents and those identifying as moderately to highly religious displayed greater resilience.

This aligns with other studies demonstrating that internal belief systems alongside fewer caregiving responsibilities enhance adaptive capacity to stressors (Zaman et al., 2023; Saleh and Al-Khatib, 2024).

A substantial proportion of participants exhibited a heightened sense of danger, with pharmacists and laboratory technicians reporting the highest scores. This may reflect their comparatively less exposure to occupational stress prior to the escalation of events, in contrast to physicians and nurses, who are more accustomed to high-stress environments. Notably, only a small percentage of participants had prior training in crisis management, which may partially account for the elevated danger perception observed. Furthermore, a heightened sense of danger was positively associated with previous exposure to security escalations, while participants with children aged 11–17 demonstrated comparatively lower danger scores, potentially reflecting a protective desensitization effect or altered risk perception associated with parenthood.

An unexpected finding was that non-users of personal support talks and group activities demonstrated higher resilience scores in unadjusted analyses. This apparent paradox likely reflects selection effects rather than ineffectiveness of support services. Individuals experiencing greater distress may have been more likely to seek psychosocial support, thereby creating a reverse association between service utilization and resilience.

Additionally, access to and quality of available services during active conflict may have been limited, informal or inconsistently structured. It is also possible that stigma surrounding mental health support influenced who engaged with such services. Therefore, these findings should not be interpreted as evidence that psychosocial interventions are ineffective. Rather, they highlight the need for systematic evaluation of service quality, accessibility and timing in conflict settings.

From an MHPSS perspective, the high levels of perceived danger and low resilience observed in this study highlight the urgent need for structured psychosocial support interventions for healthcare workers in conflict settings. Traditional in-person services may be difficult to implement during active conflict due to insecurity and infrastructure disruption. In this context, electronic mental health and psychosocial support (eMHPSS) approaches such as tele-counseling, mobile-based interventions and remote peer support have emerged as scalable and accessible alternatives (Ahmed and Heun, 2024). These modalities have shown promise in improving access to care in humanitarian settings, although their effectiveness depends on digital access, cultural acceptability and implementation quality.

Longitudinal designs would be required to clarify the causal relationship between service utilization and psychological resilience.

The statistically significant – but weak – positive correlation between sense of danger and resilience was unexpected. While prior studies have suggested that perceived danger generally undermines psychological coping capacity (Tadesse et al., 2023. Our findings suggest that in high-stress environments, a heightened awareness of risk may coexist with stronger adaptive strategies, particularly for those with prior exposure or formal training. Alternatively, this may indicate the development of hypervigilant coping behaviors, a phenomenon documented in trauma-exposed populations (Stewart et al., 2022). A study on the mental health impact of war in Libya found that hyperarousal is associated with multiple factors, including female gender, residence in major conflict zones and higher educational attainment. These findings align with broader evidence from North African and Palestinian contexts, which highlights that trauma in prolonged conflicts is not a single event but an ongoing lived experience, shaped by fragile institutions, disrupted services and limited access to psychosocial support (Abusafia et al., 2026; Raheel et al., 2026).

The positive association between resilience and sense of danger may appear counterintuitive, as resilience is often viewed as protective against psychological distress. However, resilience does not imply reduced perception of threat; rather, it reflects the ability to adapt and cope despite recognizing danger. Individuals with higher resilience may possess greater situational awareness and acknowledge existing threats more realistically, particularly in unstable or conflict settings. This interpretation aligns with previous evidence linking psychological distress indicators such as depression and anxiety with future-oriented anxiety, suggesting that heightened awareness of risk can coexist with adaptive coping mechanisms. In prolonged conflict environments, perceiving danger may represent an adaptive response that promotes vigilance and preparedness, while resilience enables individuals to continue functioning despite these stressors (Snoubar et al., 2025).

Seen through this lens, our findings were great perceived danger exists alongside sustained attendance and measurable resilience, which reflect the lived reality of healthcare workers in chronic conflict settings. In such environments, heightened awareness of threat may represent not only psychological strain but also a necessary, adaptive vigilance that enables individuals to continue functioning despite ongoing instability.

## Practical implications and policy section

The findings of this study have important implications for both health system resilience and workforce sustainability in conflict-affected settings. The high levels of perceived danger and the predominance of low resilience among healthcare workers underscore the urgent need for structured psychosocial support and mental health services tailored to frontline staff. The lack of crisis management training among the majority of participants points to a critical gap in preparedness that could be addressed through targeted training initiatives. Additionally, these insights can inform institutional policies related to staff deployment, safety protocols and supportive supervision in high-risk environments. Strengthening these systems not only safeguards the well-being of healthcare providers but also ensures continuity of care during periods of instability. Future research should explore the long-term psychological impacts of repeated exposure to insecurity and assess the effectiveness of resilience-building interventions.

This study highlights that sustaining healthcare delivery during armed conflict requires protecting the psychological well-being of healthcare workers, not only maintaining infrastructure and supplies.

1. Integration of mental health into emergency planning

The high levels of perceived danger and low resilience indicate the need to integrate mental health and psychosocial support (MHPSS) into hospital emergency response plans. Routine psychological screening, confidential counseling services and structured peer-support programs should become standard components of wartime preparedness.

1. Mandatory crisis management training

Since crisis management training was associated with better attendance and resilience, it should be institutionalized across all healthcare cadres. Incorporating safety protocols and simulation-based preparedness into both hospital policy and medical curricula can strengthen adaptive capacity during prolonged instability.

1. Workforce protection and retention strategies

Attendance was influenced by injury, caregiving responsibilities and hospital assignment. Policies should therefore include safe accommodation, secure transportation, flexible scheduling and risk allowances to reduce preventable absenteeism and burnout.

1. Health system resilience

Workforce resilience is central to health system resilience. Policymakers must recognize frontline staff mental health as a strategic priority in fragile settings. Long-term monitoring and evaluation of resilience-building interventions are essential to ensure sustainability.

Overall, protecting healthcare workers’ psychological and physical safety is fundamental to maintaining continuity of care during humanitarian crises.

## Limitation

There are many limitations to this study. Although all operating governmental hospitals in Khartoum state were included in this study, this research was conducted in only one state during the war. Since other states were also in conflict, this may limit the generalizability of results to the whole country.

Moreover, this study was conducted amid the missile attacks on Omdurman, which might have influenced the participation rate due to internet connection issues and high workload on HCPs amid the attacks.

An important methodological limitation of this study is the potential for survivor bias. Our sample included only healthcare workers who remained employed and operational in the selected hospitals during the study period. Individuals who fled the conflict, were severely traumatized, physically incapacitated, dismissed or killed could not be represented. Consequently, the psychological burden reported in this study likely underestimates the true impact of the conflict on the broader healthcare workforce. Those most severely affected may be systematically absent from the sample. Additionally, although standardized tools were used, their cross-cultural validity in the Sudanese conflict context may be limited, as cultural differences in the expression and perception of psychological distress could affect the accuracy and interpretation of the findings.

Furthermore, the relatively high attendance rate observed may not necessarily reflect psychological resilience, but rather constrained alternatives, economic necessity, professional obligation or lack of evacuation options. Therefore, findings should be interpreted as reflecting the condition of the surviving and actively working subgroup of healthcare workers, rather than the entire pre-war healthcare workforce.

Additionally, the self-reporting technique used might have influenced participants to present themselves in more socially acceptable manners, even though the questionnaire was anonymous.

Finally, the convenient sampling on the hospital level may induce selection bias despite stratified random sampling on the institutional level.

## Conclusions

This study highlights the critical psychological strain on healthcare workers during the ongoing conflict in Sudan. While a strong sense of duty maintains high work attendance, the prevalence of low resilience and a high perceived sense of danger suggests that this commitment is being sustained at a significant personal cost. These findings underscore an urgent need for the integration of Mental Health and Psychosocial Support (MHPSS) frameworks, including digital eMHPSS interventions, to protect the wellbeing of the frontline workforce. Future longitudinal research is required to further investigate predictors of resilience and the long-term impact of wartime stressors on healthcare delivery.

## Supporting information

10.1017/gmh.2026.10242.sm001 Mohamed et al. supplementary material Mohamed et al. supplementary material

## Data Availability

The datasets used and analyzed during the current study are available from the corresponding author on reasonable request.

## Supplementary material

The supplementary material for this article can be found at http://doi.org/10.1017/gmh.2026.10242.

## Long descriptions

### Table 1. Long description

The table consists of three columns: Variable, n, and percent.

* Age: 18 to 40 (314, 96.6 percent); 41 and above (11, 3.4 percent).

* Gender: Male (139, 42.8 percent); Female (186, 57.2 percent).

* Marital status: Single (248, 76.3 percent); Married (71, 21.8 percent); Divorced (3, 0.9 percent); Widowed (3, 0.9 percent).

* Do you have dependents in your household?: Yes (138, 42.5 percent); No (187, 57.5 percent).

* Children’s age: Less than 2 years (35, 10.8 percent); 2 to 10 (69, 21.2 percent); 11 to 17 (28, 8.6 percent); 18 plus (15, 4.6 percent); No children (206, 63.4 percent).

* Perceived religiosity: Low (21, 6.5 percent); Moderate (252, 77.5 percent); High (52, 16.0 percent).

* Nationality: Sudanese (323, 99.4 percent); Non-Sudanese (2, 0.6 percent).

* Living arrangements: Alone (40, 12.3 percent); In dorms (13, 4.0 percent); With family (272, 83.7 percent).

* Monthly income in S D G: Less than 500,000 (277, 85.2 percent); 500,000 to 1,000,000 (40, 12.3 percent); More than 1,000,000 (8, 2.5 percent).

Navigate back to Table 1..

### Table 2. Long description

The table consists of three columns: Variable, n (count), and % (percentage).

Occupation:

- Consultant: 4 (1.2%)

- House officer: 90 (27.7%)

- Lab technician: 21 (6.5%)

- Medical officer: 83 (25.5%)

- Nurse: 62 (19.1%)

- Pharmacist: 16 (4.9%)

- Registrar: 44 (13.5%)

- Specialist: 5 (1.5%)

Field of specialty for consultants, specialists, and registrars:

- Internal medicine: 8 (2.5%)

- General surgery: 8 (2.5%)

- Orthopedic surgery: 4 (1.3%)

- Pediatrics: 11 (3.5%)

- Obstetrics and gynecology: 12 (3.8%)

- Radiology or neurology: 2 (0.6%)

Hospital:

- Alnau: 111 (34.2%)

- Albuluk: 86 (26.5%)

- Alsuadi: 70 (21.5%)

- Bir Alwaldeen: 58 (17.8%)

Transportation to work:

- Public transportation: 186 (57.2%)

- Hospital shuttle services: 82 (25.2%)

- Private vehicle: 45 (13.8%)

- Walking/Bicycle: 12 (3.7%)

Crisis management training:

- Yes: 127 (39.1%)

- No: 198 (60.9%)

Navigate back to Table 2..

### Table 3. Long description

The table contains three columns: Variable, Absenteeism (full or partial) percentage (N), and No absenteeism percentage (N).

* Overall attendance: 33.23 percent (108) absent; 66.77 percent (217) not absent.

* Gender: Males show 25.2 percent (35) absenteeism versus Females at 39.2 percent (73).

* Marital status: Widowed individuals have the highest absenteeism at 100.0 percent (3), followed by Married at 45.1 percent (32), Divorced at 33.3 percent (1), and Single at 29.0 percent (72).

* Occupation: Pharmacists have the highest absenteeism at 62.5 percent (10), while Medical officers have the lowest at 24.1 percent (20). Other roles include Consultant (50.0 percent), Lab technician (47.6 percent), Nurse (40.3 percent), Specialist (40.0 percent), Registrar (34.1 percent), and House officer (26.7 percent).

* Hospital: Alsuadi has the highest absenteeism at 51.4 percent (36), while Albuluk has the lowest at 9.3 percent (8). Alnau is at 45.0 percent and Bir Alwaldeen is at 24.1 percent.

* Crisis management training: Those with training have lower absenteeism (25.2 percent) compared to those without (38.4 percent).

* Physically injured by terrorism: Those injured have higher absenteeism (50.7 percent) than those not injured (28.7 percent).

* Family member injured by terrorism: Those with injured family members have higher absenteeism (42.1 percent) than those without (24.7 percent).

Navigate back to Table 3..

### Table 4. Long description

The table contains six columns: Variable, B, S E, beta, t, and p.

* Age groups: B 5.227, S E 2.628, beta 0.109, t 1.989, p 0.048.

* Job profile: B 1.315, S E 0.377, beta 0.191, t 3.487, p 0.001.

* Level of work attendance: B 3.516, S E 0.972, beta 0.191, t 3.619, p less than 0.001.

* Hospital you are working in: B minus 1.859, S E 0.409, beta minus 0.237, t minus 4.548, p less than 0.001.

* Training in crisis management: B minus 2.644, S E 0.923, beta minus 0.149, t minus 2.863, p 0.004.

* Used personal support talks: B 3.688, S E 1.463, beta 0.139, t 2.521, p 0.012.

* Participated in group activities: B 3.986, S E 1.367, beta 0.175, t 2.917, p 0.004.

* Sense of danger: B 0.379, S E 0.094, beta 0.204, t 4.029, p less than 0.001.

Note: B is the unstandardized regression coefficient, S E is the standard error, beta is the standardized regression coefficient, t is the t-statistic, and p is the p-value.

Navigate back to Table 4..

### Table 5. Long description

The table consists of six columns: Variable, B (unstandardized regression coefficient), S E (standard error), beta (standardized regression coefficient), t (t-statistic), and p (p-value).

* The first data row for Exposure to security escalation events shows a B of minus 1.344, S E of 0.58, beta of minus 0.142, t of minus 2.317, and p of 0.021.

* The second data row for Resilience scale shows a B of 0.136, S E of 0.034, beta of 0.252, t of 4.029, and p of less than 0.001.

A note at the bottom defines the abbreviations used in the column headers.

Navigate back to Table 5..
