# Supplementary material for: Resilience, perceived danger and work attendance of Sudanese healthcare workers in war zones: the April 2023 armed conflict
Source: Glob Ment Health (Camb). 2026 Jun 18;13:e160. doi: 10.1017/gmh.2026.10242 (PMC13433333; doi:10.1017/gmh.2026.10242)
Supplement: Supplementary file 1 [file S2054425126102428sup001.docx]

**Table S1: Exposure to Terrorist Events and Use of Support Services**

| **Variable** | **n** | **%** |
| --- | --- | --- |
| **Property damaged by terrorism?** |  |  |
| Yes | 194 | 59.7% |
| No | 131 | 40.3% |
| **Physically injured by terrorism?** |  |  |
| Yes | 67 | 20.6% |
| No | 258 | 79.4% |
| **Family members injured by terrorism?** |  |  |
| Yes | 159 | 48.9% |
| No | 166 | 51.1% |
| **Exposed to security escalations at a previous workplace?** |  |  |
| Yes | 133 | 40.9% |
| No | 192 | 59.1% |
| **Used employee daycare services?** |  |  |
| Yes | 49 | 15.1% |
| No | 276 | 84.9% |
| **Used hospital-provided sleeping arrangements?** |  |  |
| Yes | 113 | 34.8% |
| No | 212 | 65.2% |
| **Used personal support from hospital staff?** |  |  |
| Yes | 39 | 12.0% |
| No | 286 | 88.0% |
| **Participated in workplace group support?** |  |  |
| Yes | 57 | 17.5% |
| No | 268 | 82.5% |
| **Used a community support center?** |  |  |
| Yes | 39 | 12% |
| No | 286 | 88% |

**Table S2: Summary of Non-Parametric Analyses of Factors Associated with Resilience Scores**

| **Variable** | **n** | **Median** | **Test Statistic** | **Z-Stat** | **p-value** | **Effect Size** |
| --- | --- | --- | --- | --- | --- | --- |
| **Gender** |  |  | *U* = 9986.50 | -3.51 | < 0.001 | *r* = 0.20 |
| Male | 139 | 27.00 |  |  |  |  |
| Female | 186 | 22.50 |  |  |  |  |
| **Parenthood** |  |  | *U* = 10415.50 | -2.26 | 0.024 | *r* = 0.13 |
| Non-Parents | 206 | 25.00 |  |  |  |  |
| Parents | 119 | 22.00 |  |  |  |  |
| **Religiosity** |  |  | *H*(2) = 10.240 |  | 0.006 | η² = 0.03 |
| Low | 21 | 17.00 |  |  |  |  |
| Moderate | 252 | 25.00 |  |  |  |  |
| High | 52 | 26.00 |  |  |  |  |
| **Occupation** |  |  | *H*(5) = 24.572 |  | < .001 | η² = 0.08 |
| Specialist/Registrar | 49 | 28.00 |  |  |  |  |
| Medical officer | 83 | 26.00 |  |  |  |  |
| House officer | 90 | 24.00 |  |  |  |  |
| Pharmacist/Lab technician | 37 | 22.00 |  |  |  |  |
| Nurse | 62 | 20.00 |  |  |  |  |
| Consultant | 4 | 10.00 |  |  |  |  |
| **Hospital** |  |  | *H*(3) = 32.486 |  | < 0.001 | η² = 0.10 |
| Alnau | 111 | 28.00 |  |  |  |  |
| Albuluk | 86 | 26.00 |  |  |  |  |
| Alsuadi | 70 | 23.50 |  |  |  |  |
| Bir Alwaldeen | 58 | 19.00 |  |  |  |  |
| **Crisis Management Training** |  |  | *U* = 10006.50 | -3.11 | 0.002 | *r* = 0.17 |
| Received | 127 | 28.00 |  |  |  |  |
| Not Received | 198 | 23.00 |  |  |  |  |
| **Work Attendance** |  |  | *U* = 14296.50 | 3.24 | 0.001 | *r* = 0.18 |
| Full | 217 | 27.00 |  |  |  |  |
| Partial/None | 108 | 21.00 |  |  |  |  |
| **Support Talks** |  |  | *U* = 7179.00 | 2.913 | 0.004 | *r* = 0.16 |
| Not Used | 286 | 25.00 |  |  |  |  |
| Used | 39 | 19.00 |  |  |  |  |
| **Group Activity** |  |  | *U* = 9468.00 | 2.844 | 0.004 | *r* = 0.16 |
| Not Participated | 286 | 25.00 |  |  |  |  |
| Participated | 57 | 20.00 |  |  |  |  |

*Note.* *Md* = Median; *n* = sample size; *U* = Mann-Whitney U test statistic; *z* = z-score; *H* = Kruskal-Wallis H test statistic; *r* = effect size correlation coefficient; η² = eta-squared.

**Table S3: *Summary of Non-Parametric Analyses for Sense of Danger Scores***

| **Variable** | **n** | **Median** | **Test Statistic** | **Z stat** | **p-value** | **Effect Size** |
| --- | --- | --- | --- | --- | --- | --- |
| **Occupation** |  |  | *H*(5) = 20.338 |  | .001 | η² = .06 |
| Pharmacist/Lab technician | 37 | 18.00 |  |  |  |  |
| Specialist/Registrar | 49 | 15.00 |  |  |  |  |
| House officer | 90 | 14.00 |  |  |  |  |
| Medical officer | 83 | 13.00 |  |  |  |  |
| Nurse | 62 | 13.00 |  |  |  |  |
| Consultant | 4 | 8.50 |  |  |  |  |
| **Hospital** |  |  | *H*(3) = 8.484 |  | .037 | η² = .03 |
| Albuluk | 86 | 15.00 |  |  |  |  |
| Alnau | 111 | 14.00 |  |  |  |  |
| Alsuadi | 70 | 13.00 |  |  |  |  |
| Bir Alwaldeen | 58 | 13.00 |  |  |  |  |
| **Child 11-17 Yrs** |  |  | *U* = 5217.50 | 2.24 | .025 | *r* = .12 |
| Without Child | 297 | 14.00 |  |  |  |  |
| With Child | 28 | 12.00 |  |  |  |  |
| **Escalation Exp.** |  |  | *U* = 11118.00 | -1.99 | .047 | *r* = .11 |
| Exposed | 133 | 14.00 |  |  |  |  |
| Not Exposed | 192 | 13.00 |  |  |  |  |
| **Group Activity** |  |  | *U* = 9157.50 | 2.39 | .017 | *r* = .13 |
| Not Participated | 268 | 14.00 |  |  |  |  |
| Participated | 57 | 12.00 |  |  |  |  |

*Note.* *Md* = Median; *n* = sample size; *U* = Mann-Whitney U test statistic; *z* = z-score; *H* = Kruskal-Wallis H test statistic; *r* = effect size correlation coefficient; η² = eta-squared.
