# Supplementary material for: Resilience, perceived danger and work attendance of Sudanese healthcare workers in war zones: the April 2023 armed conflict
Source: Glob Ment Health (Camb). 2026 Jun 18;13:e160. doi: 10.1017/gmh.2026.10242 (PMC13433333; doi:10.1017/gmh.2026.10242)
Supplement: Peer review history

# Reviewer report: Review: Resilience, perceived danger and work attendance of Sudanese healthcare workers in war zones: the April 2023 armed conflict — R0/PR2

**DOI:** 10.1017/gmh.2026.10242.pr2

Introduction and Background

1. The Introduction is currently disorganised and would benefit from restructuring as follows:

o Begin with a general overview of armed conflicts worldwide and their overall consequences for mental health and trauma.

o Then provide examples from other conflict-affected settings with similar contexts, such as, Iraq, Syria, and Gaza.

o Narrow the focus specifically to Sudan, including:

• A brief historical overview of the conflict,

• The current situation, and

• Its general mental health impact.

o After that, focus specifically on the impact on healthcare workers.

o Instead of stating “to our knowledge this is the first study,” please include a clear paragraph on the gaps in the existing literature, followed by the significance of the present study.

o Clearly state the study objectives and research questions at the end of the Introduction.

2. To strengthen the background, please incorporate recent literature on the mental health impact of war and conflict on populations and on health systems, including:

o https://www.tandfonline.com/doi/abs/10.1080/13623699.2024.2411651

o https://link.springer.com/article/10.1186/s12982-024-00161-6

o https://pmc.ncbi.nlm.nih.gov/articles/PMC10938685/

o https://www.medrxiv.org/content/10.1101/2021.09.27.21264178v1.full

Study Design and Methods

3. In the Study Design and Setting section, please indicate which reporting guideline or checklist was followed (e.g., STROBE).

4. Please justify why these four hospitals were selected. Were they chosen through purposive sampling, convenience sampling, or another method?

5. The Methods section has important limitations and requires additional detail. Please include:

o A clear description of the sample recruitment process,

o Detailed participant characteristics,

o The war happened for 2 years why people recuitred hwo have one momths work, I guess? I didn’t undertsna from this one moth on method. Please clarfy

o A dedicated paragraph on measurement instruments, including:

• Whether the tools are standardised and validated,

• How they were translated, and give the details of this process.

• And the Cronbach’s alpha in the current study, for all scales.

6. Please add full details of the data collection procedures and the eligibility criteria for participants.

7. Please clearly specify the statistical analyses used for each research question.

8. Please include ethical considerations, including:

o Institutional Review Board (IRB) approval, and

o Procedures for obtaining informed consent from participants.

Results and Discussion

9. Because the research questions and hypotheses are not clearly stated, the Results section currently appears random and difficult to follow. Please:

o Clearly define the research questions and/or hypotheses,

o Reorganise the Results section accordingly,

o And ensure that Results and Discussion are clearly separated.

10. In the Discussion section, the authors should:

• Systematically interpret and explain the findings,

• Compare results with previous studies,

• And discuss the implications of similarities and differences.

Implications, Recommendations, and Policy Relevance

11. It is essential to include clear recommendations and policy implications, particularly in the context of active conflict. For example:

• Strategies for supporting healthcare workers,

• The role of digital mental health services during war and conflict,

• And approaches to maintaining mental health care delivery in insecure settings.

The following Lancet article may be helpful for framing this section:

https://www.thelancet.com/journals/lancet/article/PIIS0140-6736(25)00492-1/fulltext

Limitations and Conclusion

12. Please add a dedicated Limitations section addressing:

• Methodological constraints,

• Sampling issues,

• Measurement limitations,

• And potential sources of bias.

13. The Conclusion should be short, clear, and concise, and should:

• Avoid repeating large parts of the Results and Discussion,

• Focus only on the key findings and their main implications.

# Reviewer report: Review: Resilience, perceived danger and work attendance of Sudanese healthcare workers in war zones: the April 2023 armed conflict — R0/PR3

**DOI:** 10.1017/gmh.2026.10242.pr3

**Authors:** Ejemai Eboreime

**Affiliations:** Psychiatry , Dalhousie Medical School, Canada

This manuscript makes an important contribution to understanding healthcare workforce resilience in conflict settings. The core finding that workers maintain high attendance despite severe psychological distress, has significant humanitarian implications. However, several key issues require attention.

1. Temporal Inconsistency. Your manuscript states data collection occurred in “January–March 2025” for events from “April 2023.” This creates fundamental interpretive problems:

Are you measuring resilience during active conflict or residual effects 2 years later?

The CD-RISC-10 assesses current psychological state, not retrospective states during 2023

This distinction fundamentally changes how readers should interpret all findings

Please clarify if this a retrospective assessment of 2023 trauma conducted in 2025, or were data collected during ongoing conflict.

2. Your positive correlation between perceived danger and resilience contradicts established literature. While you briefly mention “hypervigilant coping,” this finding deserves substantial theoretical engagement:

Could this reflect post-traumatic growth or survivor bias (only those with both traits remained)? Does this challenge your conclusion that high danger perception is uniformly problematic? The weak correlation (r=0.178, 3% variance) may be a cross-sectional artifact Consider expanding discussion of this finding with theoretical grounding and alternative explanations.

3. Survivor Bias is Inadequately Addressed. Your sample only includes workers who stayed and remained functional. Those who fled, were severely traumatized, injured, or killed cannot be represented. This means that your “low resilience” findings likely underestimate the true psychological toll. Findings cannot generalize to the full healthcare workforce during the conflict. The high attendance rate may reflect lack of alternatives rather than resilience. Add explicit discussion of survivor bias in limitations and interpret findings accordingly.

4 Non-users of support services showed higher resilience. This contradicts your policy recommendations for psychosocial support. Possible explanations (selection effects, ineffective services, reverse causation) need exploration.

# Reviewer report: Review: Resilience, perceived danger and work attendance of Sudanese healthcare workers in war zones: the April 2023 armed conflict — R0/PR4

**DOI:** 10.1017/gmh.2026.10242.pr4

**Authors:** Nadia Amro

**Affiliations:** Palestine Polytechnic University, Palestine, State of

the topic is great

please (clarify non-users of personal support talks )

# Reviewer report: Review: Resilience, perceived danger and work attendance of Sudanese healthcare workers in war zones: the April 2023 armed conflict — R0/PR5

**DOI:** 10.1017/gmh.2026.10242.pr5

The authors present an important and timely topic within both local and international contexts, particularly in light of armed conflicts and ongoing wars in many parts of the world. Such conditions have profound effects on healthcare workers in terms of psychological resilience, perceived risk, and continuity of work. The current study aims to explore levels of resilience, risk perception, and presenteeism among healthcare workers in a conflict setting, using validated measurement tools and clearly articulated ethical procedures. This represents a notable strength of the study.

However, several critical issues require careful attention before the manuscript can be considered for publication, as outlined below:

First: Issues related to sample size and distribution

The study reports calculating an initial sample size and then applying a finite population correction, resulting in an adjusted sample size of approximately (n ≈ 322). However, the distribution of the sample across hospitals (132, 86, 103, 66) does not align with this adjusted figure, as the total exceeds the corrected sample size and appears closer to a proportional allocation based on the unadjusted initial sample. Furthermore, the final sample size reported in the results (n = 325) adds further ambiguity regarding the actual procedure followed. It is therefore essential to clarify whether the allocation was based on the initial or adjusted sample size, how rounding or intentional oversampling was handled, and to ensure consistency of sample size figures across all sections of the manuscript.

Second: Inconsistencies in the description of the sample and professional categories

The study states in the study population section that a specific group of physicians (house officers) was excluded, yet this group appears in the statistical tables of the results. In addition, the data collection procedures sometimes refer to inviting “physicians” only to participate, while the results clearly show participation from multiple professional categories. These inconsistencies require careful revision and precise redefinition of the study population, as well as inclusion and exclusion criteria, to accurately reflect the actual participating sample.

Third: Contradictory directions of results between univariate and multivariate analyses

The study reveals inconsistencies between univariate analysis results and multivariate regression findings, particularly regarding the role of crisis management training, participation in psychological support sessions or group activities, and exposure to security incidents. In some sections, these variables are interpreted as being associated with higher resilience or perceived risk, while regression models indicate opposite directions. This likely reflects issues related to variable coding, reference category selection, or interpretation of statistical coefficients. A comprehensive review of the regression models is therefore required, with consistent interpretation across tables, narrative text, and the executive summary.

Fourth: The need to deepen the discussion and link findings to comparable Arab/regional contexts

Despite the importance of the findings, the discussion section requires clearer strengthening through engagement with Arab and regional contexts characterized by protracted conflicts, fragile resources, and overlapping professional roles among healthcare workers under pressure. This contextualization is not a cosmetic addition; rather, it enhances the scientific value by: (1) interpreting findings within social and institutional structures similar to the study setting, (2) clarifying the generalizability of results to comparable Arab contexts, and (3) highlighting the study’s contribution relative to global literature often produced in relatively stable environments.

I recommend explicitly supporting the discussion with references to Arab and Middle East and North Africa (MENA) literature on trauma, war, and social–health interventions. Relevant examples that merit integration into the discussion—organized around themes such as prolonged trauma, social support, access barriers, and future anxiety—include:

Raheel, M. F. S., Snoubar, Y., & Yarboua, E. A. (2025). Cost of War: Exploring Trauma in the North African Countries Affected by Armed Conflict. Social Work, swaf043.

Abusafia, B., Snoubar, Y., Turan, Z., Durat, G., & Almadhoun, R. (2025). Exploring Postpartum Depression among Palestinian Women in Genocide: A Feminist Perspective on Social Support and Health Access in Gaza. Affilia, 08861099251387632.

Snoubar, Y., Abusafia, B., & Şen Tepe, Ç. (2025). Addressing War-Related Trauma and Future Anxiety through Social Work: Evidence from a Quantitative Study of War-Affected Youth.

Integrating these works will add interpretive depth to the discussion and make it more compelling for an international readership, demonstrating that the findings are not isolated but aligned with emerging regional knowledge trajectories.

Fifth: Inconsistency in describing levels of resilience

The results indicate that approximately 41% of participants exhibit low resilience, while the discussion states that more than two-thirds of the sample suffer from low resilience. This discrepancy requires correction and harmonization of figures and descriptions between the results and discussion sections.

Sixth: Adding a “Practical Implications and Policy Recommendations” section before the conclusion

I recommend adding a standalone section before the conclusion titled, for example, Practical Implications and Policy Recommendations. This section would translate the findings into actionable recommendations, such as:

Designing or strengthening crisis management training and institutional psychological support programs within hospitals.

Policies aimed at protecting healthcare workers and reducing “risk burden” to improve presenteeism.

Feasible social and professional support interventions tailored to low-resource conflict settings.

Including this section would enhance the practical utility of the study for policymakers and hospital administrators and increase its citation potential.

# Editor report: Recommendation: Resilience, perceived danger and work attendance of Sudanese healthcare workers in war zones: the April 2023 armed conflict — R0/PR6

**DOI:** 10.1017/gmh.2026.10242.pr6

**Affiliations:** Director, National Institute of Mental Health, Czech Republic

Please address all reviewers' comments.

# Editor report: Decision: Resilience, perceived danger and work attendance of Sudanese healthcare workers in war zones: the April 2023 armed conflict — R0/PR7

**DOI:** 10.1017/gmh.2026.10242.pr7

**Affiliations:** London School of Hygiene & Tropical Medicine, United Kingdom of Great Britain and Northern Ireland

No accompanying comment.

# Reviewer report: Review: Resilience, perceived danger and work attendance of Sudanese healthcare workers in war zones: the April 2023 armed conflict — R1/PR9

**DOI:** 10.1017/gmh.2026.10242.pr9

The manuscript addresses an important and timely topic, and the revised version shows some improvement; however, several major issues remain insufficiently addressed and require further revision before the manuscript can be considered for publication.

The Introduction has been partially improved but still lacks a clear and coherent structure. It does not fully follow a logical progression from global evidence on armed conflict and mental health, to regional examples, and finally to the specific context of Sudan and healthcare workers. The background remains somewhat fragmented, and the manuscript would benefit from a clearer synthesis of global and regional literature. In particular, examples from other conflict-affected settings such as Iraq, Syria, Gaza, and Ukraine are only briefly mentioned and not critically integrated. The Sudan context, although introduced, still lacks sufficient depth in terms of historical overview, current conflict dynamics, and the broader mental health burden.

A key issue that remains unresolved is the absence of a clearly articulated research gap. The manuscript does not include a dedicated paragraph explicitly identifying what is missing in the existing literature and how the present study addresses this gap. In addition, the study aim and research questions, although now included, are not appropriately positioned. These should be clearly stated at the end of the Introduction, not within the Methods section, and should logically follow from the identified gaps in the literature.

Furthermore, the manuscript does not adequately incorporate the recommended recent and high-impact literature. Several previously suggested references, including recent studies on conflict-related mental health and health systems, have not been integrated. This significantly weakens the scholarly grounding of the manuscript and limits its relevance within the current global mental health evidence base. The authors are strongly encouraged to incorporate and critically engage with the following key references that remain missing: https://link.springer.com/article/10.1186/s12982-024-00161-6, https://pmc.ncbi.nlm.nih.gov/articles/PMC10938685/, https://www.medrxiv.org/content/10.1101/2021.09.27.21264178v1.full, These should not only be cited but meaningfully integrated to strengthen the background and discussion.

With regard to reporting standards, although the authors state that STROBE guidelines were followed, this is not sufficient. STROBE is not merely a checklist to be mentioned; it is a reporting framework that should be systematically applied throughout the manuscript. The authors should ensure that all relevant STROBE items are adequately addressed, including transparent reporting of study design, sampling, variables, bias, and statistical methods, and should provide an appropriate citation for the STROBE guideline.

The Methods section has improved and now includes clearer descriptions of sampling, eligibility criteria, and data collection procedures. However, some important clarifications are still needed. The inclusion criterion requiring at least one month of work during the conflict remains insufficiently justified, particularly given that the conflict has been ongoing for approximately two years. The rationale for this threshold should be more clearly explained. In addition, although validated instruments were used, the manuscript does not address issues of cultural adaptation, language, or contextual validity, which are particularly important in conflict-affected populations.

Another important issue concerns the alignment between research questions, statistical analysis, and results. The manuscript currently does not clearly demonstrate how specific statistical analyses correspond to each research question. The Methods section should explicitly state which statistical tests were used to answer each research question, and the Results section should be structured accordingly, clearly indicating where and how each research question is addressed. This alignment is essential for clarity and scientific rigor.

The Results section is better structured than before, but some parts remain overly descriptive, with minor redundancy between tables and text. Greater emphasis should be placed on synthesising key findings rather than repeating numerical data.

The Discussion section has improved and includes some comparison with previous studies, as well as consideration of survivor bias; however, it still lacks strong theoretical grounding. The findings are not sufficiently interpreted within established frameworks such as trauma theory, resilience models, and Mental Health and Psychosocial Support (MHPSS) approaches, including emerging electronic mental health and psychosocial support (eMHPSS) modalities (e.g., https://www.thelancet.com/journals/lanpsy/article/PIIS2215-0366(24)00070-1/abstract), which are increasingly relevant in conflict and humanitarian settings. In addition, comparisons with other conflict settings remain limited and not systematically developed. The concept of “adaptive hypervigilance,” while interesting, is not adequately supported by existing theoretical or empirical evidence and should therefore be interpreted more cautiously.

The policy and implications section is a valuable addition and highlights relevant areas such as MHPSS integration and crisis management training; however, the recommendations remain somewhat general and would benefit from greater specificity and innovation. In particular, the manuscript does not adequately address the role of digital or remote mental health interventions, including eMHPSS, which are critical in insecure and resource-limited settings. The authors are also encouraged to strengthen this section by engaging with high-impact, policy-oriented literature, including recent publications in The Lancet on health systems and mental health in conflict settings (e.g., https://www.thelancet.com/journals/lancet/article/PIIS0140-6736(25)00492-1/fulltext), in order to better frame the implications within the broader global health policy discourse.

The Limitations section is well developed and appropriately acknowledges key issues such as survivor bias and sampling limitations. However, it should also explicitly address measurement limitations, including the use of self-report tools and potential concerns regarding cross-cultural validity.

The Conclusion is clearer and more concise than in the previous version, but some statements still overinterpret the findings. The authors are encouraged to adopt a more cautious tone and focus on the main findings and their implications without overstating conclusions.

In summary, while the manuscript has improved, significant issues remain, particularly regarding the Introduction, integration of recent literature, theoretical framing, and alignment between research questions, methods, and results. These limitations need to be addressed to meet the standards of a high-impact journal.

Overall, a further round of revision is required.

# Editor report: Recommendation: Resilience, perceived danger and work attendance of Sudanese healthcare workers in war zones: the April 2023 armed conflict — R1/PR10

**DOI:** 10.1017/gmh.2026.10242.pr10

**Affiliations:** Director, National Institute of Mental Health, Czech Republic

Please address reviewer’s comments.

# Editor report: Decision: Resilience, perceived danger and work attendance of Sudanese healthcare workers in war zones: the April 2023 armed conflict — R1/PR11

**DOI:** 10.1017/gmh.2026.10242.pr11

**Affiliations:** London School of Hygiene & Tropical Medicine, United Kingdom of Great Britain and Northern Ireland

No accompanying comment.

# Reviewer report: Review: Resilience, perceived danger and work attendance of Sudanese healthcare workers in war zones: the April 2023 armed conflict — R2/PR13

**DOI:** 10.1017/gmh.2026.10242.pr13

Dear Authors,

Thanks for addressing the comments.

Good luck

# Editor report: Recommendation: Resilience, perceived danger and work attendance of Sudanese healthcare workers in war zones: the April 2023 armed conflict — R2/PR14

**DOI:** 10.1017/gmh.2026.10242.pr14

**Affiliations:** Director, National Institute of Mental Health, Czech Republic

Thank you for successfully addressing all points raised by the reviewer and congratulations on this important study.

# Editor report: Decision: Resilience, perceived danger and work attendance of Sudanese healthcare workers in war zones: the April 2023 armed conflict — R2/PR15

**DOI:** 10.1017/gmh.2026.10242.pr15

**Affiliations:** London School of Hygiene & Tropical Medicine, United Kingdom of Great Britain and Northern Ireland

No accompanying comment.
